# Patient-specific instruments: advantages and pitfalls

**DOI:** 10.1051/sicotj/2017054

**Published:** 2017-12-11

**Authors:** Mahmoud A. Hafez, Kirti Moholkar

**Affiliations:** 1 The Orthopaedic Department, Faculty of Medicine, October 6 University, Cairo Egypt; 2 Droitwich Knee Clinic, Bromsgrove Knee Clinic and Royal Orthopaedic Hospital, Bromsgrov B60 2JL UK

**Keywords:** PSI, Logistics, Technical features, Total knee replacement, Pitfalls, Custom-made cutting guides

## Abstract

Patient-specific instruments (PSI) aim to improve the accuracy of total knee replacement (TKR) based on computer-assisted preoperative planning. In this work, the authors describe the advantages and pitfalls of PSI based on their clinical experience. The main conclusion of this work is that PSI has direct impact on the logistical and technical features of TKR with some advantages and pitfalls.

## Introduction

Accurate placement of the knee prosthesis is a critical step in total knee replacement (TKR), and few degrees of mal-alignment can lead to failure. Conventional arthroplasty procedures have high success rate; however, they involve several technical steps (sizing, adjustment of alignment and rotation, then bone cutting) which are dependent on each other and thus could lead to accumulation of errors [1–3]. Patient-specific instrument (PSI) is one of computer-assisted orthopaedic surgery techniques that aim to perform virtual surgery based on preoperative imaging (CT or MRI). In TKR using PSI, preoperative planning is done for sizing, alignment and bone cutting, followed by designing the cutting blocks and fabricating the femoral and tibial templates, which are placed precisely over the distal femur and the proximal tibia in a best-fit fashion [4,5].

Patient-specific jigs and related technology is adopted by surgeons with intention to replace conventional TKR and computer navigation, while improving accuracy of implant sizing and positioning, saving time and improving the overall outcomes [6–8].

In this review, the authors discuss advantages and pitfalls of PSI based on their experience. Although all features could be considered as pitfalls, they carry hidden advantages. Each author presents his own experience with PSI. The first author (MAH) uses in-house manufactured PSI which is not related to implant companies. This is called hospital-based PSI where scanning, 3D planning of surgery, production of PSI, sterilization/packing and surgery are done in the same one workplace (the hospital). The second author (KM) has an experience with commercially available PSI provided by implant's companies.

## Advantages and pitfalls

### Cost

Patients need to have a preoperative MRI or CT scan prior to the surgical procedure. This additional cost ranges between £400–800 per scan based on different centres and countries [9] in addition to the cost of PSI itself. In today's global economic climate, some state-provider services can barely afford this additional expenditure. Some insurance companies pass this expense onwards to the members (patients). It is quite important from the surgeon's perspective to make patients aware of this fact to avoid confusion and resentment as an unexpected bill in the pre-/per- or postoperative phase of knee replacement surgery if desired [10]. On the other hand, conventional instruments are very expensive (50–150 USD) and they need sophisticated sterilization measures.

The UK Government has invested £200 million in 2 years on modernizing the sterilization facilities. In addition, each implant company has specific instruments and technical steps which elevate the cost of the procedure: sometimes the instruments are given for free but the overall cost offsets towards the cost of the prostheses [11]. In the first author's experience, the use of hospital-based PSI has the advantage of low cost: 25 USD for CT scan; 200 USD for MRI in addition to the cost of the templates themselves, while saving the cost of the conventional instruments [12].

### Scanning

Surgeons should pay attention to the technology that is used to acquire preoperative images which is linked to accuracy of jig placement. The patients are subjected to the radiations if preoperative CT scan is the preferred choice of preoperative imaging [13]. An MRI scan is preferred by some service providers to reduce this radiation dosage; however, both modalities of preoperative imaging have their own advantages and disadvantages. CT scan is easy to use with fewer limitations than MRI (i.e., difficult segmentation, contraindications with the presence of pacemaker, implants and obesity) [14]. Commercially available MRI-based systems have average 6-week interval from the time of performing MRI until PSI production and delivery to the hospital, and this would have the risk of anatomical changes resulting from daily activities and loading during the waiting period. These anatomical changes also carry the risk of wrong segmentation and subsequent malpositioning of the PSI [15].

CT-based software are more user-friendly and image segmentation can be done automatically, so the surgeon can do the planning/designing, unlike MRI-based software where designing can only be done by experienced technicians for manual segmentation of the images [16].

### Time

Time is divided into three: waiting time, delivery time and operative time. There can be approximately 3 to 8 week delay between patients being listed for surgery until the real-time surgery takes place [17]. This time delay is due to the need for preoperative imaging (CT or MRI Scan), time for the images to be transferred to the PSI jig/implant manufacturers, images to be agreed as acceptable by the manufacturer engineers, preoperative planning carried out by the engineers, plan to be verified or altered by the operating surgeon, manufacturing of the jigs (based upon the final surgeons accepted plan), transportation of the jigs to the operating hospital and sterilization of the jigs (in some cases the jigs are dispatched sterile). This time can be reduced but cannot be eradicated as the jigs are based upon accurate and acceptable images and the surgeon's interpretation of the proposed plans made by the engineers who are based off site (sometimes in different countries). Surgeons need to beware that in a private practice setting, offering patients a date for operation within few weeks is impossible if this technology is to be adopted. In hospital-based PSI system, waiting time could be reduced due to the elimination of logistics and time wasted for outsourcing. The delivery time is usually 1–2 weeks but it can shorten to 3–4 days in urgent cases. Reduction of theatre time is very commonly used reason for choosing patient-specific jigs in knee replacement surgery [18]. Though this factor can be quite a motivating factor, the surgeon should be aware that during the learning curve the time required is likely to be more than his own specific surgery time (approximately 20–30 minutes); in addition, if the tibial jigs were about to produce erroneous results, the time it takes to discard the jigs and go back to the conventional extra-/intramedullary jigs produces an overall increase in time taken to surgery. Operative time relies to great extent on the surgeon's skills and experience in addition to tourniquet time and anaesthesia [19]. The surgeon is governed by the time of utilizing the conventional instruments, the setting time of the cement, the time for assembly and attachment of the jigs and fixtures as well as the time for sizing, alignment, rotation and the level of bone resection. The mean operative time for PSI in comparison to conventional TKA was found to be shorter by 24 minutes which is advantageous in high-volume hospitals [20].

### Surgical learning curve

Patient-specific jigs have been used in dental surgery for some time. The technology to manufacture these jigs is still in its infancy with reference to accurate placement of jigs on the bony landmarks. Currently, accurate placement of the jigs demands denuding the soft tissues of the bone on which these jigs are to be placed. In a surgical setting, it is not desirable to detach all soft tissues of the bone to make the jigs fit better [21]. If MRI scan has been used as a preoperative imaging tool, cartilage thickness can introduce inaccuracies during planning, manufacturing or placement of the jigs. The tibial jig is the weakest link in PSI technology and care should be taken to check, assess the jig placement and the resultant cut. This is particularly the case with almost all of the patient-specific jigs with reference to the proximal tibia jig. This poses a danger of malpositioning of the jig thereby resulting in erroneous cuts in all three planes. The authors from their own experience feel that the learning curve should involve approximately 10 cases. Current methods of training aim to familiarize junior orthopaedic surgeons and nurses with TKR instrumentation systems, which vary among surgeons and hospitals.

### Lack of verification tool

Conventional navigation can be destined to <1 degree accuracy with reference to implant positioning and bony cuts. Unfortunately, if the jigs were placed inappropriately or there was movement between the accepted positioning and the definitive cut position, the verification step in navigation makes the process extremely user-friendly. Changes can be introduced before a definitive cut is carried out if the final position was not accurate or within the desired limit of acceptance [22]. Minor malalignment or malpositioning is impossible to the sight of the human eye and resultant erroneous cuts can be made if this positioning issue is not spotted before the cuts are made. The final position only becomes visible after postoperative X-rays are carried out in the radiology room. In an ideal world, the verification by means of navigation in the first 10 cases would be ideally suited to help the surgeon's learning curve to understand the tolerance of individual implant jigs and their manufacturing processes. This can also help the learning surgeon to make up his/her mind with regards to the usefulness of this technology as if the specific implant manufacturer's PSI jigs do not produce accuracy to a comparable level of computer navigation, its use in modern surgery is questionable. Some manufacturer's PSI tibial jig inaccuracy can range between 60% to an appalling 70% [3]. The femoral jig usually has a higher degree of accuracy. The authors strongly recommend that the surgeon pays utmost attention to the tibial jig and the cut position.

### Accuracy

Coronal alignment may impact clinical outcome and arthroplasty survivorship. A retrospective study (Level III evidence) evaluating 150 primary knee arthroplasty reported that knee arthroplasty with PSI restoring the mechanical axis had a similar number of outliers as conventional instrumentation with both groups having more varus outliers than knee arthroplasty with PSI restoring kinematic axis which had more valgus outliers. Malalignment of more than 3° varus or valgus to the mechanical axis in TKR is associated with early implant failure [4]. Some reports on sagittal plane accuracy have shown that patient-specific jigs do not improve accuracy in knee arthroplasty and the use of this technology is impractical as the procedure needs to be either modified or abandoned with some frequency due to the frequency of inaccurate and poor fitting tibial jigs [5]. However, in a laboratory study [18], a navigation system was used to measure the position of PSI showing that the mean error was average 0.67° (maximum 2.5°) while mean error in bone cutting was 0.32 mm (maximum 1 mm). Friedman test was used for quantative analysis and revealed significant overall agreement between the observers (*P* < 0.05). Kendall concordance coefficient was high, indicating a considerable interobserver agreement for all measured parameters except femoral cutting level. An intraobserver variation test showed significant agreement (*P* value <0.003) and the concordance coefficient was very high [18]. In terms of femoral rotation accuracy, internal rotation of the femoral component has been associated with pain, stiffness and instability [4]. A study on tibial slope and femoral component probation measured by intraoperative navigation had shown that surgeons in conventional TKR may not be able to recognize up to 10° knee flexion secondary to flexed femoral and tibial components, thus the femoral implant may become rotated internally. In PSI, the stem and keel are prepared through corresponding hole and slit on the top of the tibial cutting guide, thus determining the rotation of the tibial implant. The position of the leg (e.g., 10° of knee flexion and 20° of external to 25° of internal rotation) would significantly alter the measurements and determine limb alignment [22]. In hospital-based technique for PSI, the aim is to get the surgeon in control of the process. Therefore, complex cases and decisions could be done by the surgeon and not the technicians who currently perform planning in company-based PSI.

### Patellar kinematics

Post-operative anterior knee pain and patellar maltracking is one of the most common complications after TKA [6,7]. Rotational and sagittal component alignment has significant influence on patellar kinematics [8]. Clinical studies on conventional TKA have shown that component malpositioning may lead to wear and loosening which would in turn result in patellar instability and subsequent failure. Positioning the tibial cutting guide is a crucial step as it is positioned over the tibial plateau and closely related to the patellar tendon insertion, so clearance of soft tissues in this area must be done very carefully. To avoid pain and patellar maltracking, the position of the cutting guide as well as the implant must be verified based on surface matching, making sure that they are in best fit [14].

### Soft tissue balancing

Recently, there has been a drive from some implant manufacturers to introduce soft-tissue balancing capabilities with implants. This could be achieved as the tibial cut tolerances are still within accuracy level of less than 60% [22]. Soft-tissue balancing on an inaccurately cut tibia may result in poor outcomes and possibly early revision surgery. Soft tissue balancing with inaccurate cuts will not only result in malalignment but also poor soft tissue balancing based on erroneously done cuts, thereby producing extremely poor results. The uptake off jigs with soft tissue balancing ability has not been great which only reflects that the analytic knee reconstructive surgeons are very carefully analysing this technology before doing their surgeries [9]. In PSI, the cutting guides could be removed and trial implants could be used to adjust soft tissue balance for the mediolateral and flexion extension plane. In addition, the surgery requires minimal collateral ligaments or retinacular release.

## Discussion

A systematic review reported that majority of studies did not show an improvement in overall limb alignment when patient specific implants was compared with standard instrumentation. Mixed results were seen across studies with regard to the prevalence of alignment outliers when patient specific implants were compared with conventional cutting blocks with some studies demonstrating no difference, some showing an improvement with PSI and a single study showing worse results with PSI. The studies demonstrated mixed results regarding the influence of PSI on operative times. The reduced operative times were not uniformly observed and the accuracy of preoperative planning generated by PSI manufacturers was found lacking often leading to multiple intraoperative changes, thereby disrupting the flow of operation and negatively impacting efficiency. PSI has not clearly been shown to improve overall surgical efficiency or the cost effectiveness of knee joint arthroplasty [13].

**Figure 1:**
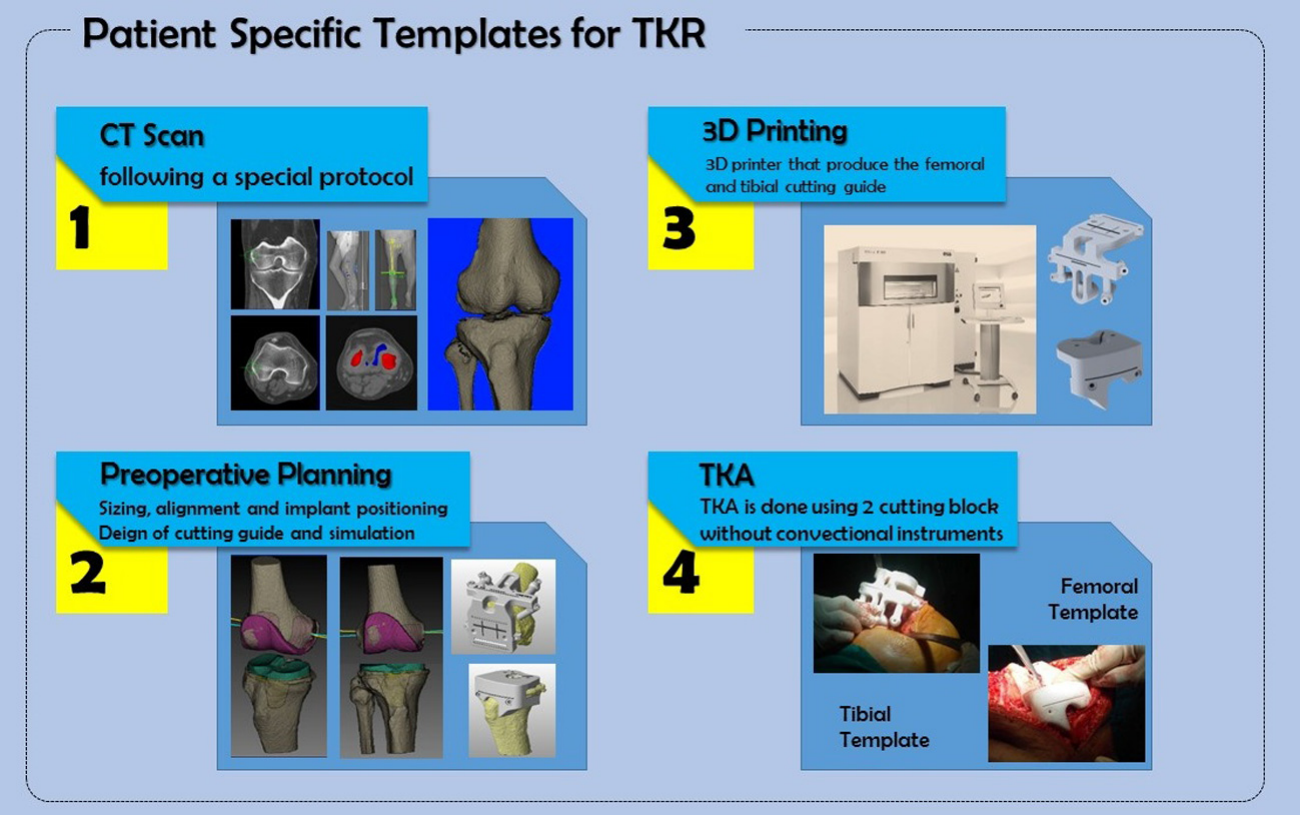
Hospital-based system for PSI.

For cost reduction and accelerated workflow, the first author (MAH) currently uses a hospital-based system for PSI. The early results of this work have shown significant waive of logistical costs which had a direct impact on the overall expenses of the procedure, that is, the cost of the production of PSI was less than £250, in addition to £25 for CT scan. The concept of hospital-based PSI means that the surgeon and the hospital are in control of PSI process by doing all steps; that is, imaging, modelling, 3D printing, sterilization and surgery (Figure 1), where transportation expenses and transferring management are eliminated.

The accuracy and reliability of the PSI were assessed on 45 TKAs (16 cadaveric and 29 plastic knees) and compared to conventional instrumentations (PFC, DePuy, Johnson and Johnson). All operations were finalized with PSI without resorting to conventional instrumentations or IM perforation [14]. In addition, computer analysis of randomly selected CT scans for PSI mean error of alignment and bone resection within 1.7° and 0.8 mm (maximum 2.3° and 1.2 mm, resp.), these results were compared to conventional techniques that had errors more than 3° [15].

It is worth mentioning that prolonged operative time increases the risk of contamination as well as the risk of developing disturbed normal anatomy (e.g., patellar dislocation or joint subluxation). This could in turn prolong the rehabilitation time, hospital stay and recovery time. Prolonged operative time could also prolong tourniquet time with subsequent risk of infection and vascular complications [20]. There is overwhelming intraoperative information in navigation and robotics especially in extremely obese patients, and in case of fragile bone or metallic implant which acts as artifact. PSI could be firstly practiced by the surgeon on plastic knee models fabricated according to patient's own CT scans so the results of the surgery could be foreseen on real patients; thus, the surgeon could position PSI to validate the accuracy, the level and the inclination of bone cutting resection so operative time would be reduced.

Another noticeable advantage of PSI is the lower rate of blood loss, blood transfusion and subsequent potential infection. Although blood transfusion is related to other factors such as age, type and degree of deformity and other comorbidities, PSI has theoretical advantage over conventional TKA that it eliminates opening the medullary canal; in addition, it shortens the operative time [21].

## Conflicts of interest

The authors declare that they have no conflicts of interest in relation to this article.
